# Natural firing patterns reduce sensitivity of synaptic plasticity to spike-timing

**DOI:** 10.1186/1471-2202-14-S1-P304

**Published:** 2013-07-08

**Authors:** Michael Graupner, Srdjan Ostojic

**Affiliations:** 1 Center for Neural Science, New York University, New York, USA; 2 Group for Neural Theory, Laboratoire de Neurosciences Cognitives, Ecole Normale Superieure, Paris, France

## 

Synaptic plasticity is sensitive to both the rate and the timing of pre- and postsynaptic spikes. In experimental protocols used to induce plasticity, the imposed spike trains are regular and the relative timing between every pre- and postsynaptic spike is fixed. This is at odds with natural firing patterns observed in the cortex of intact animals, where cells fire irregularly and the timing between pre- and post-synaptic spikes varies.

To investigate synaptic changes elicited by in vivo-like irregularly firing neurons at different rates and realistic correlations between pre- and post-synaptic spikes, we use numerical simulations and mathematical analysis of synaptic plasticity models. We concentrate on a calcium-based model [1], and further consider a voltage-based model [2] and a spike-timing based model [3]. To allow for comparison, all models are fitted to plasticity results obtained in vitro [4].

We show that standard stimulation protocols overestimate the influence of spike-timing on synaptic plasticity. Using a simple modification of regular spike-pair protocols, we allow for neurons to fire irregularly. Such irregular spike-pairs reduce the amplitude of potentiation and depression obtained by varying the time difference between pre- and postsynaptic spikes. This protocol allows us to quantify the relative effects of firing rate and timing in natural firing patterns, and to predict changes induced by an arbitrary correlation function between pre- and post-synaptic spikes. We show that spike correlations change synaptic plasticity at low firing rates in all models; whereas their influence becomes negligible at high firing rates for the calcium-based model but remains significant for the other two models. Our findings yield predictions for novel experiments and help bridge the gap between existing results on synaptic plasticity and plasticity occurring under natural conditions.
